# Transient Photoluminescence Reveals the Dynamics of Injected Charge Carriers in Perovskite Light-Emitting Diodes

**DOI:** 10.1021/acsami.4c19379

**Published:** 2025-01-29

**Authors:** Karim Elkhouly, Marius Franckevičius, Vidmantas Jašinskas, Andrius Gelžinis, Iakov Goldberg, Robert Gehlhaar, Jan Genoe, Paul Heremans, Vidmantas Gulbinas

**Affiliations:** † IMEC, Kapeldreef 75, 3001 Leuven, Belgium; ‡ ESAT, KU Leuven, Kasteelpark Arenberg, 3001 Leuven, Belgium; § Center for Physical Sciences and Technology, Saulėtekio av.3, 10257 Vilnius, Lithuania; ∥ Institute of Chemical Physics, Faculty of Physics, Vilnius University, Sauletekio av. 9-III, 10222 Vilnius, Lithuania

**Keywords:** perovskite, light-emitting diodes, photoluminescence, electroluminescence, roll-off

## Abstract

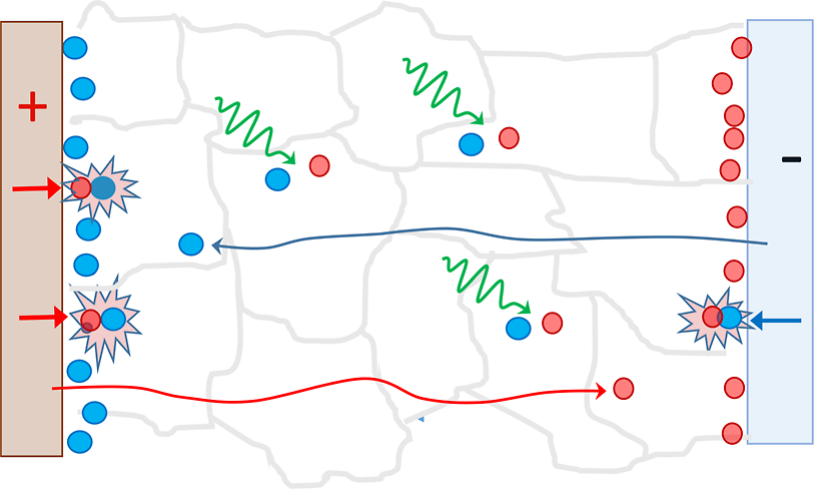

Understanding the dynamics of injected charge carriers is crucial for the analysis of the perovskite light-emitting diode (PeLED) operation. The behavior of the injected carriers largely dictates the external quantum efficiency (EQE) roll-off at high current densities and the temperature dependence of the EQE in PeLEDs. However, limitations such as sample capacitance and external circuitry hinder precise control of carrier injection rates, making it challenging to directly track the dynamics of individual carriers. Here, we explore the recombination dynamics of injected charge carriers in a small-grain methylammonium lead iodide (MAPI) PeLED pumped at high current densities by investigating the dynamics of additional carriers photogenerated by ultrashort optical pulses. We show that photogenerated charge carriers predominantly recombine in a geminate fashion within a single perovskite grain. Conversely, recombination between photogenerated and injected carriers is rare, even at current densities up to 100 A/cm^2^, due to the spatial separation caused by the internal electric field, which confines injected carriers near opposite electrodes. This spatial separation is a key mechanism behind the EQE roll-off in PeLEDs, with reduced carrier mobility at lower temperatures, mitigating this effect by weakening carrier localization and electron–hole separation.

## Introduction

Perovskite light-emitting diodes (PeLEDs) have emerged as a promising technology for next-generation display applications. Metal halide perovskite active layers are characterized by unique properties such as high charge carrier mobilities, high radiative recombination rates, and narrow emission line widths.^1−3^ This is in addition to the ease of processability, substrate compatibility, and facile fabrication methods. To date, high-performance PeLEDs with external quantum efficiency (EQE) > 10% have been demonstrated in the blue,^4,5^ green,^6,7^ red,^8^ and near-infrared ranges,^9−11^ demonstrating the viability of this technology. Furthermore, big strides were achieved in terms of increasing device lifetime, where stable PeLEDs with a half-lifetime *T*_50_ > 1000 h were demonstrated.^12^

Despite these advances, the maximum EQE of PeLEDs is commonly achieved at low current densities (*J*) < 100 mA/cm^2^, followed by a severe EQE roll-off at higher *J*.^13,14^ The origin of EQE roll-off was the subject of many studies and was attributed to different mechanisms such as Joule heating,^15^ charge imbalance,^16^ electric field-induced quenching,^17^ and Auger recombination.^18,19^ However, the assignment of the dominant mechanism varied between different research works depending on the perovskite morphology under investigation, i.e., three-dimensional (3D), quasi-two-dimensional (quasi-2D), and nanocrystalline, and the choice of charge transport layers and device electrodes.

Temperature-dependent EQE-*J* measurements are an important tool to gain deeper insight into the origin of the EQE roll-off at high current densities. He et al. demonstrated a PeLED with near-unity internal quantum efficiency at 65 K.^20^ Furthermore, Xu et al. developed pulsed PeLEDs based on quasi-2D perovskites which operate at up to several tens of A/cm^2^ and showed a reduction in the EQE roll-off at lower temperatures.^21^ Moreover, Elkhouly et al. studied EQE roll-off in scaled-PeLEDs up to 3 kA/cm^2^, where a 3-fold enhancement in EQE at high *J* was demonstrated in a PeLED based on 3D morphology with small grains.^22^ Finally, a large-grain 3D perovskite layer showed nearly a 5-fold enhancement in EQE at 77 K in reference to room temperature (RT) operation (*J* = 1 kA/cm^2^).^23^ These results demonstrate that EQE roll-off in PeLEDs has a strong temperature dependence; however, the underlying physical mechanism remains unclear.

In this work, we investigate the carrier dynamics in small-grain 3D PeLEDs, providing important insights into the processes occurring in the operating devices. Identical samples have been thoroughly studied in our previous publications, where we provided information about their structure, steady-state, and pulsed operation properties.^24−27^ However, the high-time-resolution behavior of injected carriers has not been previously addressed, as sharp carrier injection is not feasible due to difficulties in generating ultrashort electrical pulses, limitations of electrical circuits, and the sample capacitance. Therefore, we attempt to tackle this by monitoring the dynamics of optically generated charge carriers. To this end, we investigate the ultrafast luminescence dynamics of the perovskite active layer when copumped with synchronized ultrashort optical pulses and submicrosecond electrical pulses. By monitoring the luminescence dynamics following optical excitation, we obtain insights into the interaction between injected and photogenerated charge carriers. Additional time-resolved photoluminescence experiments on bare perovskite films reveal the importance of carrier confinement within the perovskite grains. These experiments allow us to argue that the field-induced spatial separation of carrier clouds localizing them next to opposite electrodes is the main mechanism for the EQE roll-off at high current densities.

## Materials and Methods

### Materials

The indium tin oxide (ITO) electrodes (150 nm, 30–40 Ω per square) were DC magnetron sputtered in-house through a shadow mask on polished sapphire substrates. PbI_2_ was obtained from TCI Chemicals. Zinc acetate dihydrate, magnesium acetate tetrahydrate, tetramethylammonium hydroxide, ethyl acetate, dimethyl sulfoxide, chlorobenzene, toluene, and dimethylformamide (DMF) were purchased from Sigma-Aldrich. PolyTPD was obtained from 1-Materials. Methylammonium iodide (MAI) and benzylammonium iodide (PMAI) were ordered from Greatcell Solar Materials. All the commercial materials were used as received.

### Perovskite Film Preparation

The perovskite films were prepared according to a method reported by Rand et al.^28^ For the MAPI film, PbI_2_ was dissolved together with MAI in DMF to obtain a 0.2 M precursor solution. Then, for the small-grain film precursor, 20 mol % extra benzylammonium iodide was added to form the final MAPI precursor solutions. The perovskite films were deposited by spin coating these precursor solutions at 6000 rpm. A solvent-exchange step was performed by dropping toluene on the substrates 3.5 s after commencing spinning. The samples were then annealed at 70 °C for 5 min^27^ The fabricated small-grain films were of 40 nm thickness composed of 10–30 nm diameter grains. The large-grain films were prepared without additional benzylammonium iodide, by using an 0.4 M precursor solution and the same solvent exchange process as for the small-grain film. The prepared films were of 80 nm thickness composed of >100 nm diameter grains. This thickness was chosen to allow for the formation of pinhole-free films.

### Device Fabrication

Prepatterned sapphire substrates with circular openings in 100 nm SiN layers were prepared in-house according to our previous report.^25^ Then, following cleaning steps in acetone and IPA, 6 mg/mL PolyTPD solution in chlorobenzene was spin coated at 4000 rpm for 60 s. This was followed by thermal annealing at 150 °C for 20 min. To improve surface wettability, the PolyTPD layer was treated with an O_2_ plasma for 6 s at a power of 100 W. After that, the perovskite films were deposited in a glovebox as described above. Then, 15 mg/mL PCBM solution in chlorobenzene and the ZnMgO nanoparticle solution, prepared according to our previous work,^27^ were spin coated onto the perovskite layers consecutively at 3000 rpm and 4000 rpm, respectively. This results in an approximately 20 nm thick PCBM layer and a 20 nm thick ZnMgO layer. The devices were finished by thermal evaporation of 100 nm of Al.

### Experimental Measurements

Electrical pulses with durations ranging from 500 ns to 5 μs, adjusted according to the operation current of the device, were applied to the PeLED devices using a Tektronix AFG3101 function generator to induce electroluminescence. Simultaneously, to induce photoluminescence, the samples were excited with 80 fs light pulses at 515 nm generated by a femtosecond Yb/KGW laser (Light Conversion Ltd.). The light was focused onto the devices with a controlled delay of approximately 200 ns relative to the leading edge of the electrical pulses. The excitation intensity of the sample was maintained at approximately 0.1 μJ/cm^2^. Electroluminescence and transient photoluminescence signals were detected using a Hamamatsu streak camera operating in a single-sweep mode with a repetition rate of 500 Hz. This configuration enabled the measurement of transient photoluminescence and the detection of prezero time signals associated with electroluminescence with a time resolution ranging from several tens of picoseconds to several nanoseconds depending on the measurement time interval. A liquid helium cold finger cryostat (Janis CCS-100/204) was used for low-temperature measurements.

## Results and Discussion

### Recombination of Injected and Photogenerated Charge Carriers

We study a PeLED stack based on small-grain (10–30 nm in diameter) methylammonium lead iodide (MAPI; 40 nm thickness), sandwiched between a 15 nm PolyTPD hole transport layer and PCBM (20 nm)/ZnMgO (20 nm) electron transport bilayer. Here, PolyTPD, PCBM, and ZnMgO refer to poly[*N*,*N*′-bis (4-butylphenyl)-*N*,*N*′-bis(phenyl)-benzidine], 6,6′-phenyl-C61-butyric acid methyl ester, and magnesium-doped zinc oxide, respectively. ITO is used as a transparent bottom electrode, while Al is used as a top electrode. The performance and stability of this PeLED were previously studied both at low current density DC and high current density pulsed operation.^25,27^ The PeLED under investigation is miniaturized using a current focusing approach, by etching holes in a silicon nitride (100 nm) layer following our previous work.^25^ Here, we study a PeLED with a diameter of 200 μm.

To investigate the dynamic behavior of electrically injected and optically generated charge carriers, we pumped our PeLEDs with short subμs electrical pulses at a repetition rate of 500 Hz and synchronously applied ultrashort (∼80 fs) optical pulse excitation. Figure S1 in Supporting Information shows the time diagram of these measurements. The optical excitation pulses were applied with a delay time of 200 ns relative to the leading edge of the electrical pulses. This allowed the injection current to reach steady state following resistive-capacitive delay.^22^ Moreover, the total duration of electrical pulses was gradually decreased from 5 μs at the lowest applied voltages to 0.5 μs at the highest voltages to allow for stable and reproducible device operation and prevent irreversible PeLED degradation. Using a streak camera, we were able to observe the luminescence dynamics with picosecond time resolution. To obtain more information about the properties of the charge carriers, we performed these studies at different temperatures ranging from 100 K to RT.

**Figure 1:**
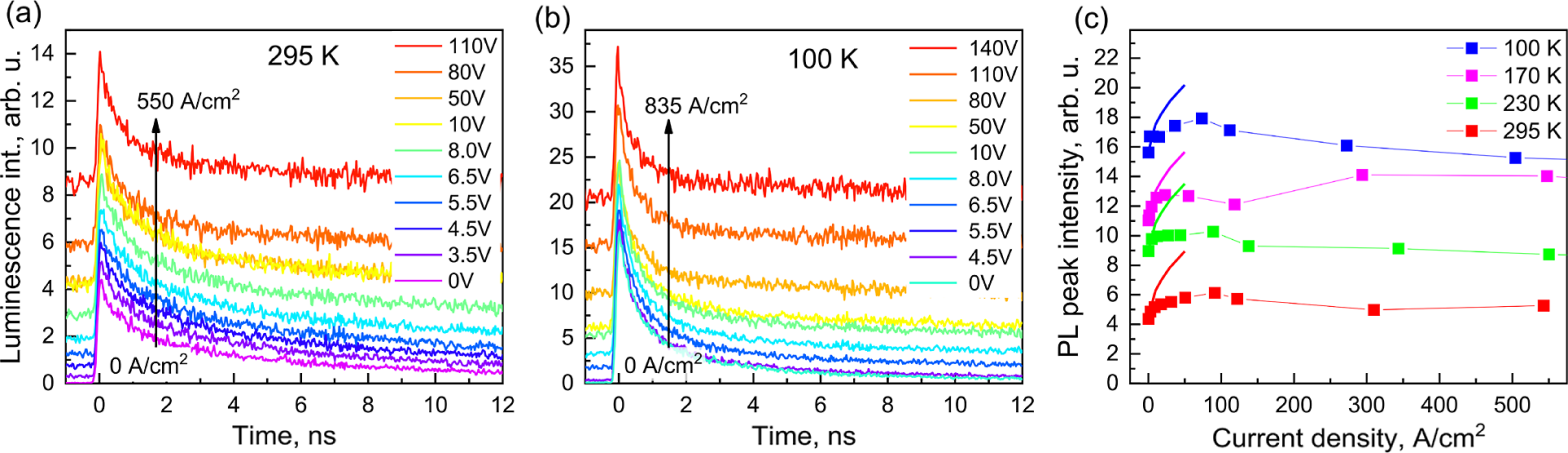
Transient luminescence dynamics of the MAPI PeLED at 295 K (a) and 100 K (b) created by simultaneous electrical and optical pumping. The PeLED was optically excited by 515 nm wavelength, 80 fs duration, and 100 nJ/cm^2^ energy density laser pulses during electrical pumping by different voltage electric pulses with duration decreasing with voltage from 5 μs to 500 ns. Signals before zero time correspond to electroluminescence; (c) shows dependence of PL_EL_ peak intensities on the injection current density obtained after subtraction of the EL signal. Smooth lines in (c) show peak intensities predicted by the classical semiconductor model. Current–voltage graphs presented in Supporting Information Figure S4a show relationship between voltage and current values at different temperatures.

Figure 1 shows the luminescence dynamics of the MAPI PeLED created by ultrashort optical pulses at different applied voltages of electric pulses at two extreme used temperatures of 295 K and 100 K. The dynamics at other used temperatures of 170 K and 230 K are shown in Supporting Information (Figure S2). Similar dependencies were also measured for the PeLED based on formamidinium lead iodide (FAPI) and are also shown in Supporting Information (Figure S3). Given that the results for the MAPI and FAPI PeLEDs are strikingly similar, we mainly focus on the MAPI PeLED.

Since electric pulses were much longer than the optical pulses, the luminescence peak created by optical excitation emerged on a background of much longer electroluminescence (EL) signal, which we observe before optical excitation at zero time. Dependencies of the EL intensity on the voltage of applied pump pulses are presented in the Supporting Information (Figure S4b).

To describe our results mathematically, we will use EL and PL for the electroluminescence and photoluminescence intensities, respectively, produced by electrical and optical pumping only, while PL_EL_ will be used to denote the photoluminescence above the electroluminescence background when photoexcitation is applied together with injection current. EL + PL_EL_, thus, corresponds to the luminescence intensity produced by both pulses. The optical excitation intensity was chosen such that the intensities of PL and EL were comparable, i.e., the photoluminescence peak without applied voltage was approximately equal to the EL intensity with an applied voltage in the range of 5–10 V.

As shown in Figure 1a,b, the total luminescence intensity is close to the sum of EL and PL intensities, thus PL_EL_ intensity only weakly depends on the injection current. However, PL_EL_ decay becomes slightly faster at high injection current densities and is also faster at low temperatures. Figure 1c shows the dependence of the PL_EL_ peak intensity on the current density more precisely. The PL_EL_ peak increases slightly with current density (*J*) up to about 100 A/cm^2^ and then saturates or even decreases slightly at stronger currents. Similar data were also obtained for the FAPI film (Figure S3). As we demonstrate below, such dependences are unexpected in the framework of the classical semiconductor model. In the simplest case, we can express the total intensity of the luminescence peak produced by both optical and electrical pumping as follows
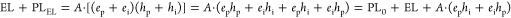
1where *e*_p_, *e*_i_, *h*_p_, and *h*_i_ are the densities of photogenerated and injected holes and electrons, respectively. Here, we introduce *A* as a proportionality constant to account for the recombination rate. Taking into account that the densities of photogenerated electrons and holes are equal (*e*_p_ = *h*_p_) and assuming balanced carrier injection (*e*_i_ = *h*_i_), we obtain
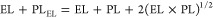
2
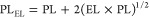
3

The cross-term 2(EL × PL)^1/2^ = *A·*(*e*_p_*h*_i_ + *e*_i_*h*_p_) should cause a significant increase in total luminescence intensity over the sum of PL and EL intensities, thus PL_EL_ should strongly increase at high current densities. For example, at 10 V voltage at RT, the EL is approximately equal to the PL peak. Then we expect the total luminescence intensity EL + PL_EL_ to be 2 times greater than EL + PL, while in fact it is greater by only about 1.3 times. At low temperatures, this relative increase is even smaller.

We modeled the PL_EL_ dependences on the current density at different temperatures using eq 3. The smooth curves in Figure 1c show that this equation describes quite well the relationship between the PL_EL_ peak intensity and current density for the low *J* values of several initial data points. This close agreement, observed at all used temperatures, strongly suggests that the conventional semiconductor model is indeed valid but only at low current densities, while some other processes are responsible for the drastic disagreement at higher current densities.

It should be noted that the cross-term 2(EL × PL)^1/2^ can also be small leading to weak PL_EL_ dependence on *J*, in the case of heavily doped perovskites, which have a large density of equilibrium carriers. Assuming we have equilibrium electrons (*e*_e_), we obtain
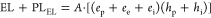
4

In the extreme case of a very large density of equilibrium electrons, when *e*_e_ ≫ *e*_i_, we obtain
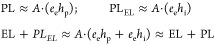
5

Thus, according to this heavily doped semiconductor model, PL_EL_ and PL should be additive values, meaning that PL_EL_ is independent of *J*. This model, however, suggests that this independence should be observed only at low densities of injected carriers, while PL_EL_ is expected to start to increase at strong *J*, when density of injected carriers exceeds density of equilibrium carriers. This is opposite the experimental data presented in Figure 1c, rendering this hypothesis unlikely.

**Figure 2:**
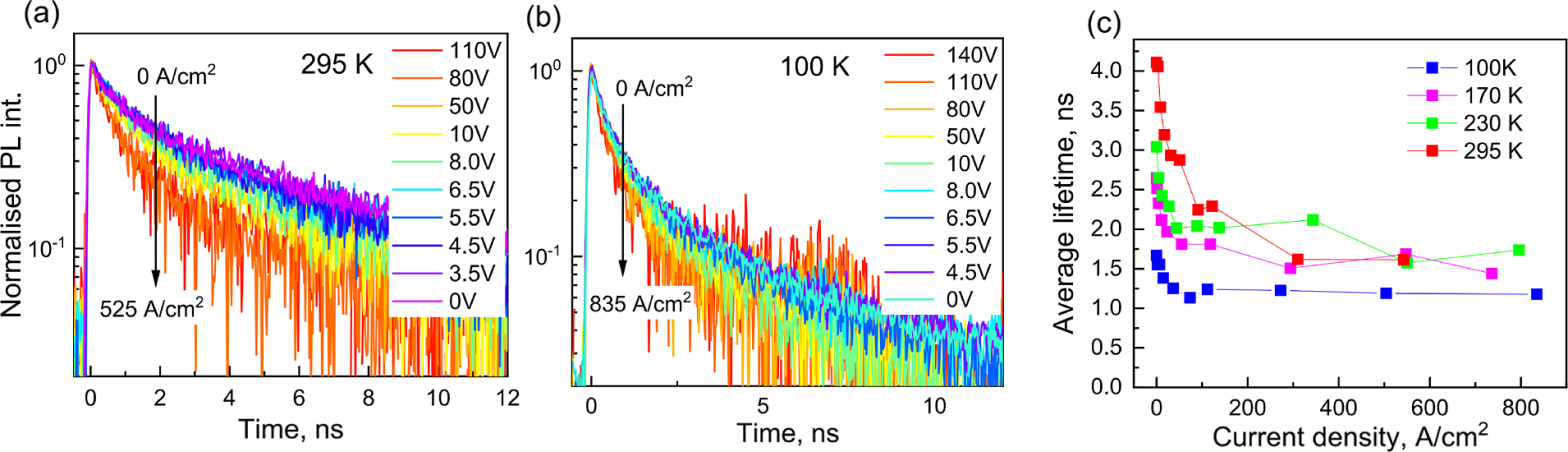
Normalized PL_EL_ kinetics at 295 K (a) and 100 K (b) obtained after subtraction of the EL background from luminescence kinetics of the MAPI PeLED simultaneously pumped by optical and electrical pulses; (c) shows average PL_EL_ lifetimes at different temperatures obtained from biexponential fitting.

Figure 2a,b shows normalized PL_EL_ kinetics at 295 K and 100 K, respectively, under different applied voltages. The PL_EL_ kinetics at other used temperatures of 230 K and 170 K are shown in Supporting Information Figure S2. The PL_EL_ kinetics at 295 K become faster at high applied voltages. This dependence is less clear at 100 K. Figure 2c shows the average lifetimes calculated as τ_ave_ = (τ_1_*a*_1_ + τ_2_*a*_2_)/(*a*_1_ + *a*_2_), where τ_1,_ τ_2_ and *a*_1_, *a*_2_ are lifetimes and their relative amplitudes obtained by fitting the PL_EL_ decay kinetics by a biexponential decay function. The average lifetime decreases by about 35% at 100 K when the current density increases to about 100 A/cm^2^. This decrease is stronger at RT; however, τ_ave_ remains stable at high currents. The decrease of the lifetime by increasing injection current agrees with the undoped semiconductor model, while the heavily doped semiconductor model predicts weak lifetime dependence on current; however, again, this weak dependence is expected at low currents.

To evaluate the actual equilibrium carrier density in our PeLED devices, we performed Mott–Schottky analysis, and the corresponding plots are presented in Supporting Information Figure S5. The equilibrium carrier densities extracted from several capacitance versus voltage measurements ranged from 2.8 × 10^16^ cm^–3^ to 5.5 × 10^16^ cm^–3^, depending on the measurement conditions. However, it should be noted that Mott–Schottky analysis can be challenging for perovskite materials due to factors such as ion migration and the presence of trap states.^29^ These issues can lead to nonideal behavior in the capacitance–voltage characteristics, causing uncertainties and variability in the extracted carrier densities. Despite these limitations, the relatively low equilibrium carrier densities obtained from the Mott–Schottky analysis support our conclusion that the doped semiconductor model is not applicable to our perovskite films. Therefore, luminescence decay kinetics leads to the same conclusions as current dependences of the PL_EL_ peak intensity. Both suggest that carrier behavior agrees with the weakly doped semiconductor model, but some additional processes diminish recombination of photogenerated carriers with injected carriers at high current densities. It should be noted that the above-described conventional semiconductor model assumes a homogeneous spatial distribution of the injected, photogenerated, and equilibrium charge carriers within the perovskite layer. It also neglects the PL quenching by the applied electric field and internal electric fields created by the inhomogeneously distributed mobile ions.

### Electronic Properties of the Small-Grain Perovskite Film

To elucidate the above-discussed discrepancies, we investigated the photoluminescence properties of a bare small-grain perovskite film on a glass substrate prepared identically as for PeLEDs and compared them with those of a similar film but prepared to be composed of relatively large ∼120 nm grains.

**Figure 3:**
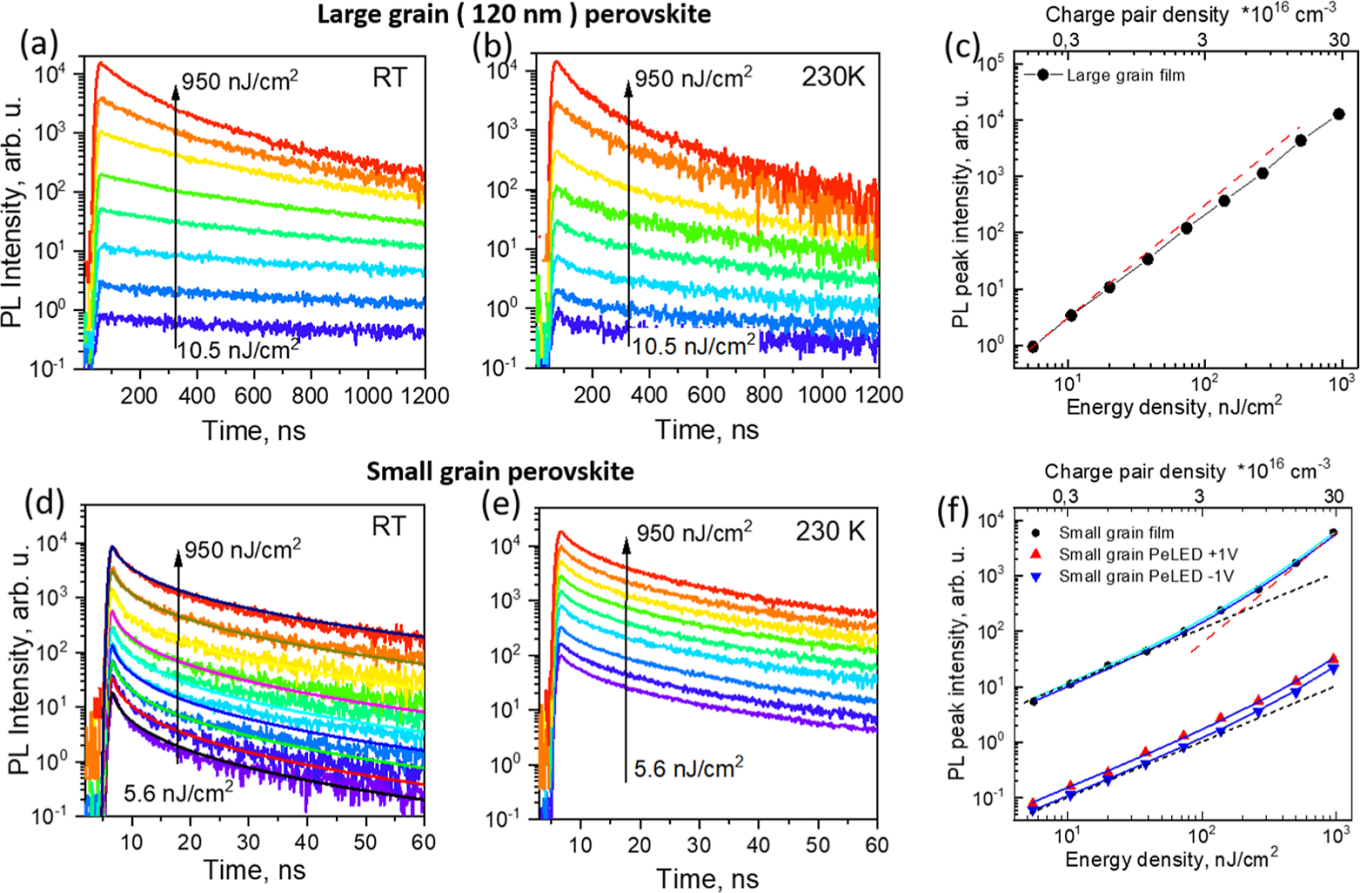
PL kinetics of large-grain (a,b) and small-grain (d,e) MAPI films on glass measured at different excitation intensities at room and 230 K temperatures. Solid lines in (d) show modeled kinetics according to the partly confined carrier model; (c) shows PL peak intensities as functions of excitation intensity for the large-grain film, dashed line indicates quadratic dependence; (f) shows similar dependencies for small-grain film (dots) and for MAPI PeLED (triangles) under forward +1 V and reverse −1 V voltages. Solid blue curves and cyan curve show fitting results by partly confined carrier and doped semiconductor models, respectively, dashed black and red lines show linear and quadratic dependencies.

Figure 3a,b,d,e shows PL kinetics of the two films at different excitation intensities and different temperatures, while Figure S6 shows the full data set of decay kinetics at RT, 230 K, 170 K, and 100 K for the two films. At RT, the PL decay of the large-grain film is independent of the excitation intensity up to about 100 nJ/cm^2^, indicating that linear Shockley–Read–Hall (SRH) recombination dominates at low excitation intensities, while at higher intensities, the decay rate increases with excitation intensity, showing that the quadratic recombination prevails. The PL peak scales quadratically with the excitation intensity and deviates slightly from this dependence at high excitation intensities (Figure 3c). This behavior is in good agreement with the previously reported carrier dynamics in MAPI^30^ and agrees with the classical undoped semiconductor model described above. At low temperatures, a fast PL decay component was present, which was particularly clear at low excitation intensities. This linear decay component is most likely caused by the carrier trapping by the shallow traps, which play only a marginal role at RT. The quadratic recombination observed at high intensities was also slightly faster at low temperatures, however less than expected,^31,32^ while the initial PL intensity slightly decreased. This is in stark contrast with the results obtained for relatively thick (submicron) perovskite layers used in the solar cells, where the initial time-resolved PL intensity increased tens of times at low temperatures,^33^ and this increase was in agreement with the experimentally observed and theoretically evaluated increase of the quadratic recombination rate coefficient.^31,32^ We speculate that the differences are related to the differences in the morphologies of the perovskite layers. According to the Langevin theory, the recombination rate is proportional to the carrier mobility, which in perovskite crystals is mainly determined by the scattering by phonons.^34^ The mobility in perovskite films is also reduced by the intergrain boundaries, which create barriers for the carrier motion, which are particularly significant at low temperatures. The role of barriers is expected to be more important in our thin film composed of relatively small (∼120 nm) flat grains. Thus, the reduced “transparency” of the intergrain barriers at low temperatures apparently partly compensates for the reduced scattering by phonons, thus limiting carrier mobility and recombination rate. Feng et al. have recently suggested that interfacial strain related to different thermal expansion coefficients of perovskite and substrate may also cause decline of the carrier mobility at low temperatures.^35^

The small-grain film shows strikingly different dependences of the PL kinetics on both excitation intensity and temperature (Figure 3d,e). At RT, the PL decay rate is nearly independent of excitation intensity in the high-intensity range but becomes faster at low excitation intensities. As the temperature decreases, this fast decay component disappears, and the kinetics becomes independent of excitation intensity. At the lowest used temperature of 100 K, the decay becomes slightly faster overall but remains independent of excitation intensity (Figure S6). The initial PL intensity (Figure 3f; small dots) increases linearly with the excitation intensity up to about 100 nJ/cm^2^ and approaches quadratic dependence only at very high intensities. This behavior disagrees with the classical semiconductor model but can be partly explained by the heavily doped semiconductor model (eq 4). If we assume *n*-doping, we can describe the PL intensity as PL = *A*·[(*e*_p_ + *e*_e_) *h*_p_]. At low excitation intensities when *e*_e_ ≫ *e*_p_, we obtain PL = *A*·(*e*_e_*h*_p_) and thus a linear increase with the density of the photogenerated holes. At high excitation intensities when *e*_e_ < *e*_p_, a quadratic dependence is expected. Modeling the PL intensity (cyan curve) gives good agreement with the experimental data, but only assuming a high equilibrium carrier density of about 5 × 10^17^ cm^–3^, which is an order of magnitude higher than that obtained from the Mott–Schottky analysis.

To additionally discard the doped semiconductor model, we have measured dependence of the PL peak intensity on the excitation intensity for the small-grain PeLED at an applied forward and reverse voltages of 1 V. The forward voltage approximately compensates the built-in electric field, and we observe very similar PL dependence on excitation intensity as in the bare film. The reverse voltage is expected to extract equilibrium charge carriers, if they are present, and to convert the PL dependence on excitation intensity to the quadratic one, as observed for the large-grain film. This approach is validated by the Mott–Schottky plots presented in Figure S5 showing almost complete saturation of the reciprocal capacitance below 0 V, which indicates complete extraction of equilibrium carriers. The applied voltage only reduced the PL intensity by about 1.5 times but did not change the dependence on excitation intensity (see Figure 3f), additionally contradicting the doped semiconductor model and additionally indicating that conventional semiconductor model cannot explain luminescence properties of the small-grain MAPI PeLED and bare film.

The morphology of the small-grain film suggests an alternative model of partially confined charge carriers (PCC) to explain the above-described contradicting data. The small-grain film consists of 10–30 nm grains surrounded by passivating ligands. In the PCC model, we assume that the photogenerated carriers are partially confined within the grain in which they were generated and only weakly interact with other carriers present in neighboring grains, at least on a short time scale until the carriers migrate to the neighboring grains. The grain boundaries are semitransparent to the charge carriers, just forming barriers to their motion. The effect of the grain boundaries therefore is expected to be less important at high temperatures when they become more transparent due to the thermal jumps. Electrons and holes may recombine radiatively only when they are located in the same grain. In this case, we expect the PL intensity to increase linearly with excitation intensity as long as no more than one charge pair is generated in an individual grain. At higher intensities, when two or more charge pairs are generated per grain, the linear PL dependence should change to a quadratic one. Thus, at high excitation intensities, this model predicts identical dependencies of the luminescence intensity and kinetics as the conventional semiconductor model; however, it explains the linear luminescence intensity dependence at low excitation intensity without assumption of the heavy material dropping.

We have calculated the PL peak intensity dependence on excitation intensity shown in Figure 3f assuming that the recombination rate is proportional to the product of the electron and hole numbers within a single grain. We calculated the probability of generation of a single or multiple charge pairs per grain at the actual excitation intensities used, with grain size serving as a single free parameter. Such a calculation gives perfect agreement with the experimental data (solid blue curves in Figure 3a), suggesting that about 6 and 3 charge pairs per grain were generated at the highest used excitation intensity of 1000 nJ/cm^2^ in the bare small-grain perovskite layer on glass and in the PeLED under study, respectively. This modeling also gives average grain sizes of about 26 nm for the bare small-grain MAPI layer on glass and 21 nm for the PeLED, which is in good agreement with the typical grain sizes obtained with the fabrication technique used.

The PCC model also explains the unusual increase in the PL decay rate at low excitation intensities (Figure 3a). Electron transfer to the neighboring grain quenches the grain fluorescence, thus accelerating the PL decay rate. But this additional quenching channel is active only at low excitation intensities when only a small fraction of the grains is excited. In contrast, at high excitation intensities, when the majority of the grains are excited, the loss of the carrier that leaves the grain may be compensated by carriers incoming from neighboring photoexcited grains. Consequently, the PL decay becomes slower at high excitation intensity, in agreement with the results presented in Figure 3a. A similar PL behavior was also reported for the films with large grains but observed on a picosecond time scale at very low excitation intensities.^36^ It was attributed to the separation of geminate charge pairs in a material where grain boundaries play a less significant role. In the case of the small-grain film under the current study, the separation is significantly slower because charge carriers must cross the grain boundaries. This model also explains the unusual difference between the PL decay kinetics at RT and those at low temperatures. The fast PL decay component at low intensities attributed to the intergrain carrier jumps disappears at low temperatures when such jumps become slower than the intragrain carrier recombination. On the other hand, PL intensity and relaxation rate increase 2–3 times by sample cooling from 230 K to 100 K (see Figure S6 in Supporting Information). In this temperature range, charge carriers remain almost confined within the individual perovskite grains. Therefore, the temperature dependence of their bimolecular recombination rate is no longer affected by the intergrain carrier transport and increases at low temperatures, in agreement with the theoretical predictions.^32^

To further validate the PCC concept, we developed a more elaborate simulation model accounting for the carrier transfer between grains to reproduce the PL decay kinetics at different excitation intensities. A more detailed description of the simulation procedure can be found in Supporting Information (Supplementary Note). We obtained perfect agreement of the simulated decay kinetics (smooth lines in Figure 3a) with the experimental data using the actual excitation intensities and grain sizes determined by the modeling of the PL peak intensity, as described above. Consequently, the PCC model perfectly reproduces both initial PL intensities and decay kinetics at different excitation intensities in the small-grain film and PeLED. The modeling results show that the initial equilibration rate that effectively describes carrier hopping between neighboring grains is *k*_0_^eq^=(0.0345)^−1^ at RT. Based on this hopping rate, we can estimate the carrier diffusion coefficient (see Supporting Information for details). Due to the time dependence of *k*_eq_, the diffusion coefficient is also time-dependent, with values decreasing from about 0.1 cm^2^·s^–1^ at 1 ps to 3 × 10^–4^ cm^2^·s^–1^ at 1 ns. Correspondingly, we determine that the carrier mobility also decreases from about 4.6 cm^2^·V^–1^·s^–1^ at 1 ps to 0.01 cm^2^·V^–1^·s^–1^ at 1 ns. These relatively low values correspond to the so-called macroscopic diffusion and mobility characterizing carrier motion at distances exceeding grain sizes and are quite expected taking into account the nanostructured perovskite morphology. At low temperatures, when the intergrain carrier hopping becomes slower, the mobility and diffusion rates apparently decrease, yet a very strong decrease is not likely, since carrier mobility should be sufficient for the drift of injected carriers across the perovskite layer and recombination.

Now let us return to the analysis of the PeLED luminescence taking into account the PCC model. This model partially explains the weak recombination of photogenerated charge carriers with injected ones. According to this model, an increase in the PL_EL_ decay rate is expected when a photogenerated charge pair and an injected charge carrier encounter in the same grain. Such encounters are rare at low injection currents, when only a small fraction of the grains contain injected charge carriers. We can estimate the density of the injected carriers from the comparison of PL and EL intensities. Considering the absorbance of the MAPI film, we estimate that a 100 nJ/cm^2^ optical pulse generated about 3 × 10^16^ cm^–3^ charge pair density. This carrier density created a similar initial peak luminescence intensity as electroluminescence created by pumping by 50 V (∼100 A/cm^2^) electrical pulses, observed in Figure 1a as luminescence before zero time. Figure S7 in Supporting Information shows the time diagram of measurements used for comparison of PL and EL intensities and carrier densities. Despite different EL and PL time dependencies, we may consider that carrier densities were identical when the EL and PL peak intensities were identical. Thus, the simplest model suggests that 100 A/cm^2^ electrical pulses cleated similar charge pair density of about 3 × 10^16^ cm^–3^ as 100 nJ/cm^2^ optical pulses. This density corresponds to about 0.3 electron and hole per single perovskite grain. Note that the actual densities of injected carriers are likely to be higher than this idealized estimate since carrier injection is hardly perfectly balanced, and the luminescence intensity produced by correlated photogenerated charge carriers should be higher than the intensity produced by the same concentration of uncorrelated injected charge carriers. Moreover, the injected electrons and holes, as we will discuss below, may be nonhomogeneously distributed and partially spatially separated reducing the EL intensity. Consequently, this rough estimation gives the lower limit of the population of grains by injected charge carriers, indicating that at least about half of the grains should contain injected electrons or holes at a pump current of 100 A/cm^2^. In the simplest case, recombination of photogenerated charge carriers in these grains should be twice as fast as that without carrier injection. Indeed, we observe about a factor of 2 shortening of the PL_EL_ lifetime at about 100 A/cm^2^ current density. However, we also expect that the PL_EL_ lifetime should further decrease at higher current densities, which was not experimentally observed. Moreover, we also expect a similar decrease of the PL_EL_ lifetime at low temperatures, but this decrease is much less significant.

### Spatial Separation of the Injected and Photogenerated Charge Carriers

The spatial separation of injected and photogenerated charge carriers along the perovskite layer thickness is the most likely reason for the reduced interaction of photogenerated and injected charge carriers. We have demonstrated in our previous works that spatial distributions of the electron and hole “clouds” along the perovskite layer thickness play an important role in determining luminescence intensity and dynamics in pulsed-pumped PeLEDs.^16,26^ Electrons and holes are injected into the perovskite layer from different sides. By drifting in the internal electric field toward each other, they recombine and create electroluminescence. We demonstrated that only a fraction of injected charge carriers recombine during their transit through the perovskite layer, while remaining fractions pass the perovskite layer and accumulate next to the opposite electrodes. Formation of the carrier-rich layers was unambiguously confirmed by the observation of the overshoot effect in perovskite LEDs and solar cells when spatially separated electron and hole clouds diffuse toward each other after termination of the electrical pump pulse and create short intense luminescence.^22,37^ Our modeling of the carrier dynamics in identical PeLEDs as investigated here showed that the thicknesses of the carrier-rich layers are of several nanometers only under applied several voltages, while carrier densities in the central part of the layer are significantly lower.^26^ This is also in line with the recently reported modeling of the carrier distribution in operating in pulsed regime 75 nm thick FAPI PeLED, where it was demonstrated that carrier density decreases approximately exponentially with distance from electrode, decreasing about 10 times at about 20 nm distance from electrode even at small 1.7 V operation voltage.^38^ On the other hand, photogeneration of charge carriers occurs approximately uniformly across the perovskite layer thickness. Thus, the majority of charge carriers are photogenerated in the perovskite region where the density of injected carriers is low. Consequently, it causes weak recombination between photogenerated and injected carriers. This recombination is significant only at very low voltages not exceeding several volts, when carrier-rich layers are relatively thick, and the injected carrier density in the central part of the perovskite layer is also substantial. This information about the dynamics of photogenerated charge carriers enables us to speculate about the properties of electroluminescence and in particular about efficiency roll-off at high applied voltages.

**Figure 4:**
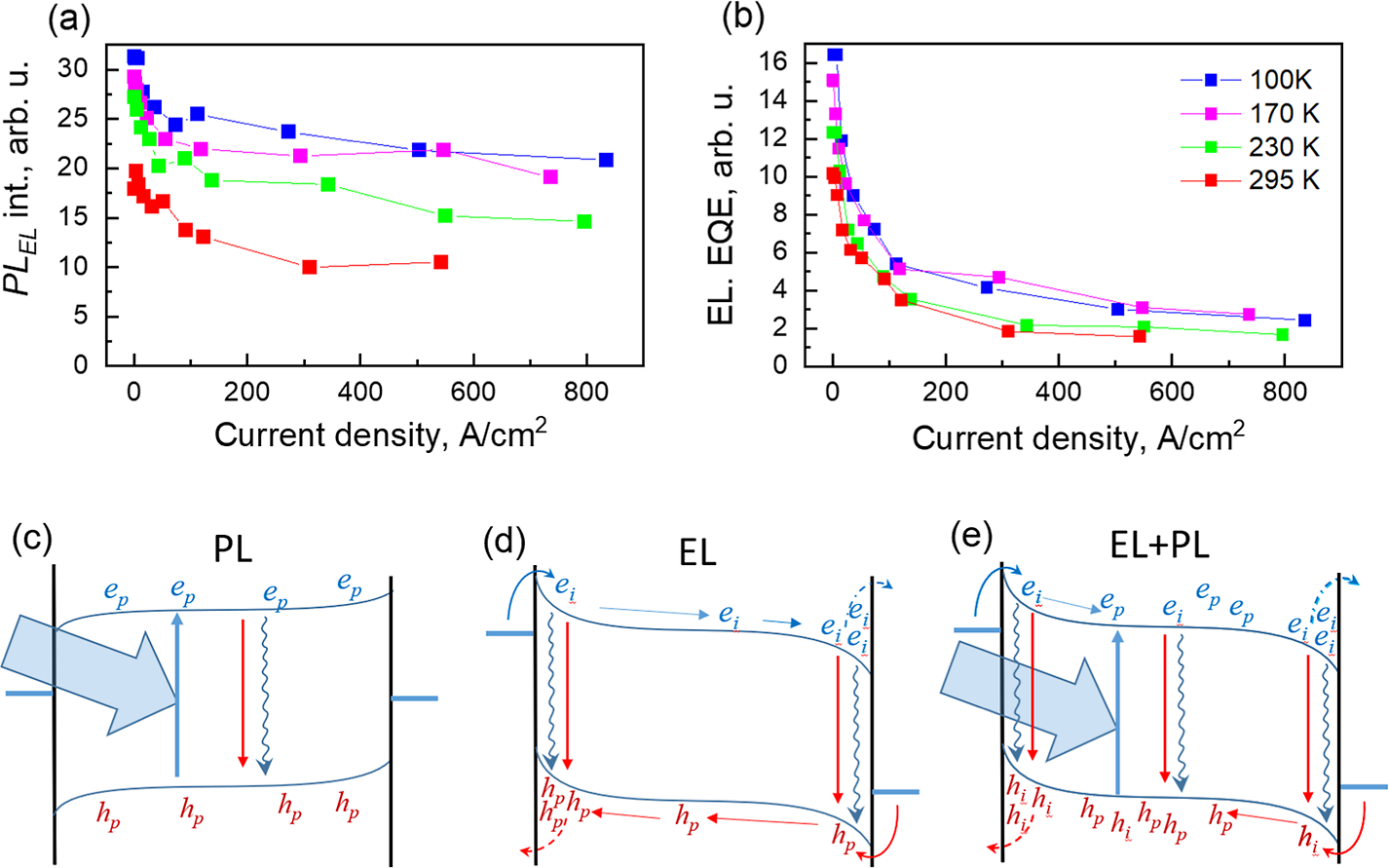
Photoluminescence intensity (a) and electroluminescence quantum efficiency (b) as functions of injection current at different temperatures; (c–e) show major electronic processes for photoluminescence, electroluminescence, and common pumping. Here, *e*_i_, *e*_p_, *h*_i_, and *h*_p_ are injected and photogenerated electrons and holes, respectively. Horizontal displacement of electron and hole positions in (b) shows separation of photogenerated electron and hole clouds by internal electric field. Plot (d) shows that fractions of injected carriers recombine near the electrodes, while remaining fractions cross the layer, accumulate near the opposite electrodes, and partly leak.

Figure 4 shows a comparison between the dependences of the PL_EL_ intensity and EQE of EL on current density. The EL EQE values were obtained from the EL background intensities of luminescence kinetics presented in Figure 1, dividing them by the current density, while the PL_EL_ intensity values were obtained by integrating PL_EL_ kinetics after subtraction of the EL background and extrapolating exponential decays to longer times. Both EL EQE and PL_EL_ intensity values decrease with current, but EL EQE decreases much stronger, about 5–8 times depending on temperature, while PL_EL_ intensity decreases only by about 1.5–2 times. Both EQE values decrease somewhat more slowly at low temperatures.

Generally, the EL EQE is determined by the competition between radiative and nonradiative recombination rates and by carrier extraction. Considering photoluminescence, carrier extraction is hardly important because the intergrain carrier hopping is relatively slow, as discussed above; therefore, carrier extraction is apparently much slower than the PL_EL_ decay. Nonradiative carrier recombination in MAPI perovskites is mainly determined by the Shockley–Read–Hall (SRH) process, which also hardly depends on the applied voltage. Therefore, the observed decrease in PL_EL_ EQE could be attributed to the field-induced spatial separation of electron and hole clouds, as shown in Figure 4e, which is more significant at high temperatures due to the faster carrier hopping. Auger recombination starts at about >10^17^ cm^–3^ carrier concentration.^18,39,40^ Such a concentration approximately corresponds to the creation of three carriers per grain. According to our estimations, such a concentration might be achievable at the strongest current densities. However, as was discussed, injected carriers are strongly localized close to electrodes and weakly interact with photogenerated carriers; therefore, Auger recombination is hardly important for photoluminescence decays at excitation intensities used for electrical/optical pumping.

Considering electroluminescence, high local densities of injected charge carriers may be achieved at high voltages when they localize close to opposite electrodes (Figure 4d). Then, they can escape through the blocking layer and recombine at the interface nonradiatively or by Auger recombination when a charge carrier is injected into the grain containing more than one opposite charge carrier. All these processes reduce the luminescence yield. At low temperatures, the intergrain carrier hoping slows considerably, and the layer crossing time increases. Thus, carrier density and their recombination also increase in the layer center, while carrier accumulation close to electrodes becomes less significant causing a less severe EQE roll-off. This EQE roll-off mechanism is supported by the previous finding that EL EQE increased at low temperatures more significantly when cooling was performed under applied positive voltage.^22^ In this case, mobile ions moving at high temperatures partly screen the electric field. Cooling fixes this ion configuration, reducing internal electric field at pulsed pumping. Thus, a lower electric field, together with reduced carrier mobilities, reduces carrier drift rates and their accumulation near electrodes. It should also be noted that the obtained results correspond to the operation of the PeLED under low-frequency pumping by short electric pulses. The situation may be significantly different under steady-state pumping when the population of trap states,^41^ ion accumulation,^26,42^ Joule heating,^15^ and electrochemical material modifications^43^ can play a crucial role and significantly affect the EL properties. We have recently demonstrated that the development of the EL intensity takes place on widely distributed time scales ranging from milliseconds to hours.^26^ Therefore, the importance of these processes in the pulsed pumping regime may also vary depending on the duration and frequency of the pump pulses.

## Conclusions

Our results demonstrate significant differences between the electronic processes in small-grain (10–30 nm) and conventional perovskites and provide a clearer understanding of the processes in the small-grain PeLEDs necessary for analysis and improvement of their performance. The charge carrier dynamics in the small-grain perovskite films are determined by the dynamics of the charge carriers within the grains (recombination and trapping) and intergrain charge transfer. The latter is comparable to the intragrain recombination and/or trapping rates at RT but significantly slows down at low temperatures. Weak recombination of injected charge carriers with additionally photogenerated charge carriers in operating PeLEDs reveals their spatial separation due to localization of injected carriers close to the opposite electrodes. This localization leads to less efficient radiative recombination, faster nonradiative recombination, and probably carrier leaking, which are the main processes responsible for the EQE roll-off observed in PeLEDs at high applied voltages. The roll-off is less significant at low temperatures when intergrain charge transfer slows, increasing the layer-crossing time and, thus, the probability of carrier recombination. Consequently, our results demonstrate that the spatial electron and hole separation both between grains and across the perovskite layer is the most important factor determining radiative recombination of injected charge carriers in the small-grain PeLEDs. It suggests that efforts to increase the PeLED efficiency and to mitigate the EQE roll-off at high current densities should be directed toward the minimization of the carrier separation across the perovskite layer.

## Supporting Information Available

Time diagram of the common electric and optical PeLED pumping, transient luminescence dynamics, Mott–Schottky analysis, PL decay kinetics, and modeling of the PL kinetics (PDF)
