# Supplementary material for: Transient Photoluminescence Reveals the Dynamics of Injected Charge Carriers in Perovskite Light-Emitting Diodes
Source: ACS Appl Mater Interfaces. 2025 Jan 29;17(6):9625–34. doi: 10.1021/acsami.4c19379 (PMC11826890; doi:10.1021/acsami.4c19379)
Supplement: Supplementary file 1 — am4c19379_si_001.pdf [file am4c19379_si_001.pdf]

Supporting information

Transient Photoluminescence Reveals Dynamics of Injected Charge Carriers in Perovskite Light Emitting Diodes

K. Elkhouly,[1,2] M. Franckevičius,[3] V. Jašinskas,[3] A. Gelžinis,[3,4] I. Goldberg,[1,2] R. Gehlhaar,[1] J. Genoe,[1,2] P. Heremans,[1,2] V. Gulbinas[3,]*

[1]IMEC, Kapeldreef 75, Leuven, 3001, Belgium

[2]ESAT, KU Leuven, Kasteelpark Arenberg, Leuven, 3001, Belgium

[3]Center for Physical Sciences and Technology, Saulėtekio av.3, Vilnius, 10257, Lithuania

[4]Institute of Chemical Physics, Faculty of Physics, Vilnius University, Sauletekio av. 9-III, Vilnius, 10222, Lithuania

[*]vidmantas.gulbinas@ftmc.lt

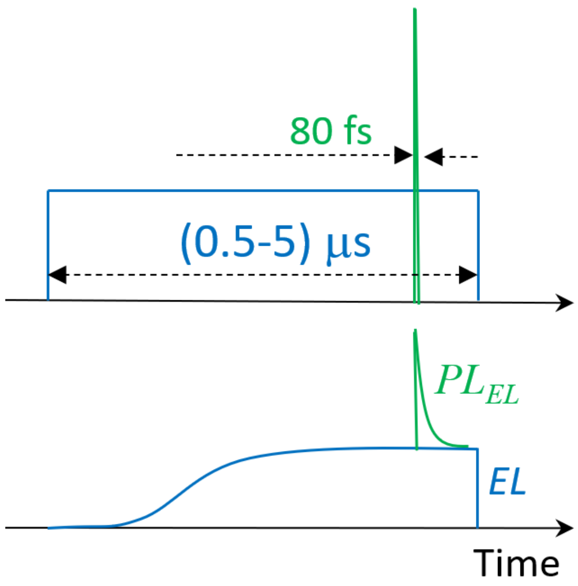

Figure S1. Time diagram of the common electric and optical PeLED pumping.

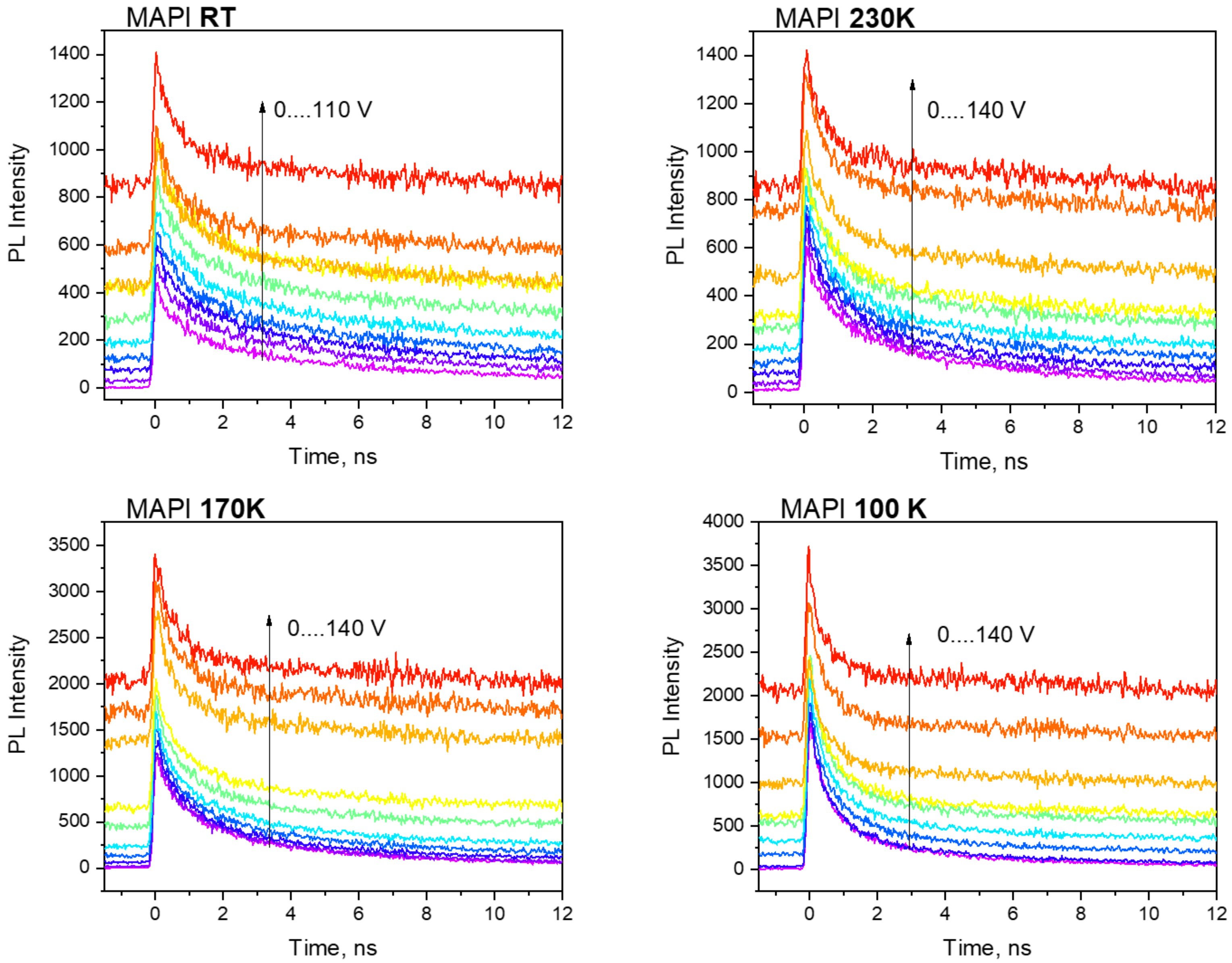

Figure S2. Transient luminescence dynamics at different temperatures of MAPI PeLED under excitation by 0.1 μJ/cm^2, 515 nm, 80 fs duration laser pulses

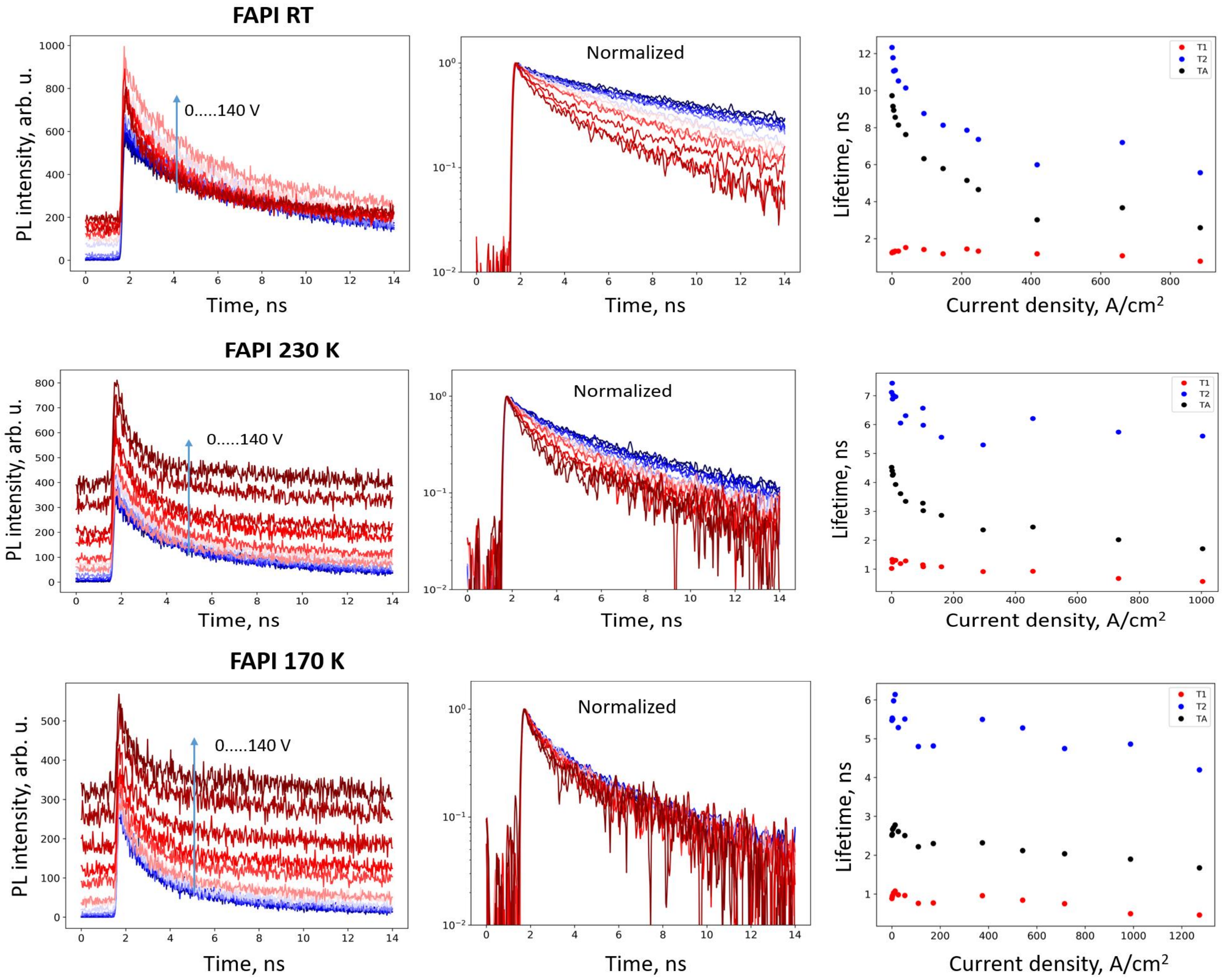
FAPI RT
PL intensity, arb. u.
0.....140 V
Time, ns
Normalized
Time, ns
Lifetime, ns
T1
T2
TA
Current density, A/cm2
FAPI 230 K
PL intensity, arb. u.
0.....140 V
Time, ns
Normalized
Time, ns
Lifetime, ns
T1
T2
TA
Current density, A/cm2
FAPI 170 K
PL intensity, arb. u.
0.....140 V
Time, ns
Normalized
Time, ns
Lifetime, ns
T1
T2
TA
Current density, A/cm2

Figure S3. Transient luminescence dynamics at different temperatures of FAPI PeLED under excitation by 0.1 μJ/cm^2, 515 nm, 300 fs duration laser pulses (left), EL subtracted and normalized (middle), relaxation times of the PL kinetics obtained by biexponential approximation and average relaxation time obtained as $TA = (\tau_1 a_1 + \tau_2 a_2)/(a_1 + a_2)$ (right).

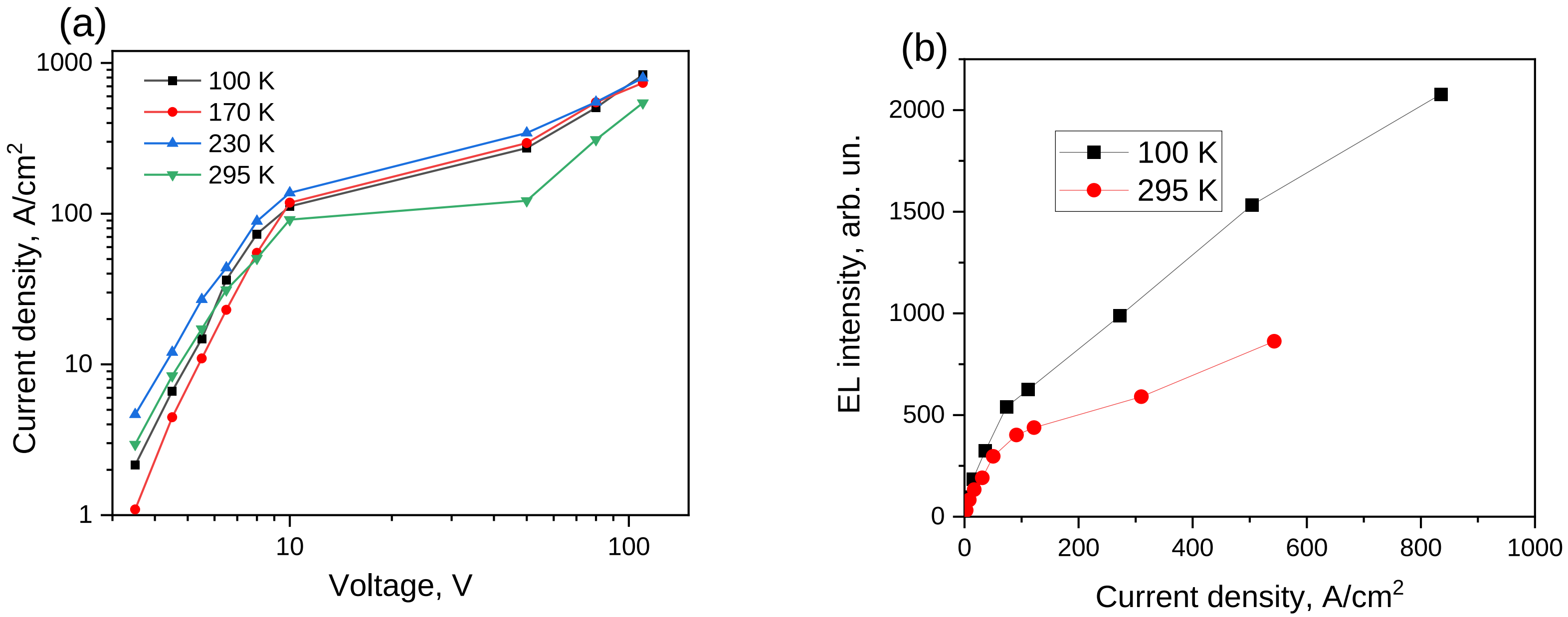

Figure S4. a) Current-voltage characteristics of the used PeLED; b) dependencies of the EL intensity on the pump current at different temperatures.

Mott-Schottky analysis

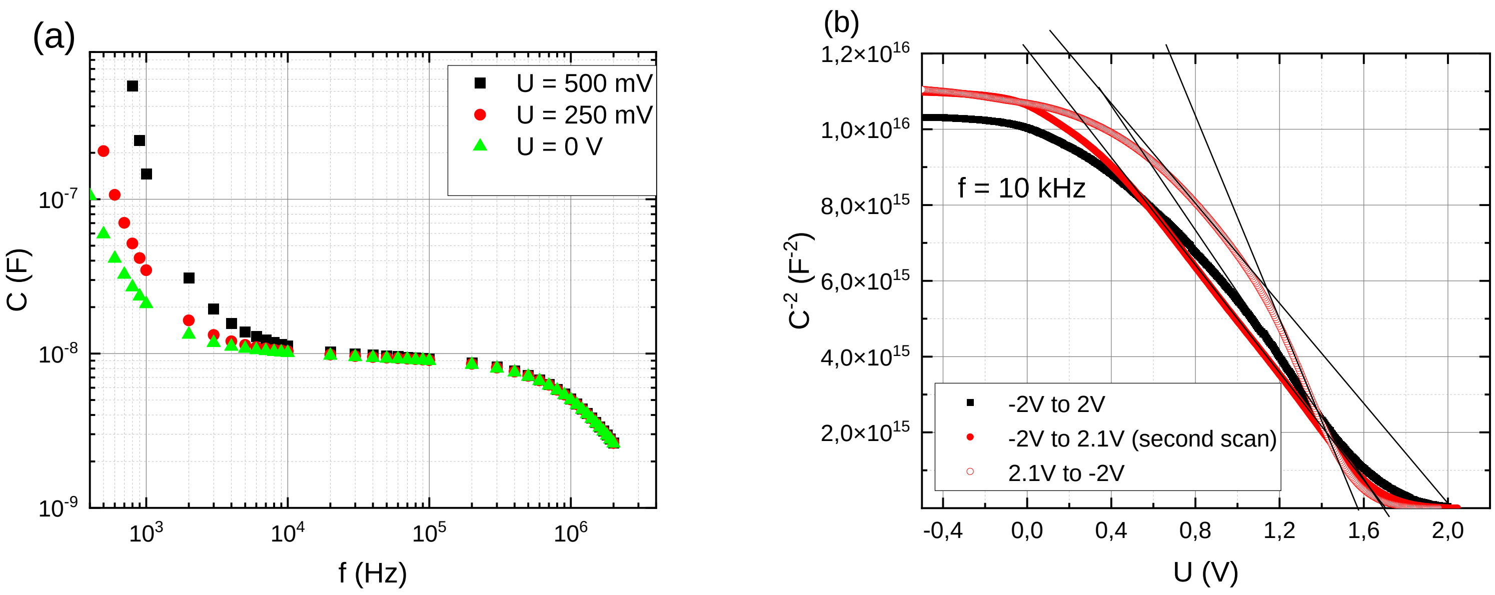

Figure S5. a) Frequency dependence of the PeLED capacitance; b) Mott-Schottky plots at 10 kHz frequency obtained for several forward and backward scans. Different shapes of the curves are attributable to different distributions of ions making the analysis and evaluation of parameters problematic and net very reliable. Straight lines show linear approximations of the curves used for the calculation of the equilibrium carrier concentrations obtained in the range between $2.8*10^{16}$ cm^{-3} and $5.5*10^{16}$ cm^{-3}.

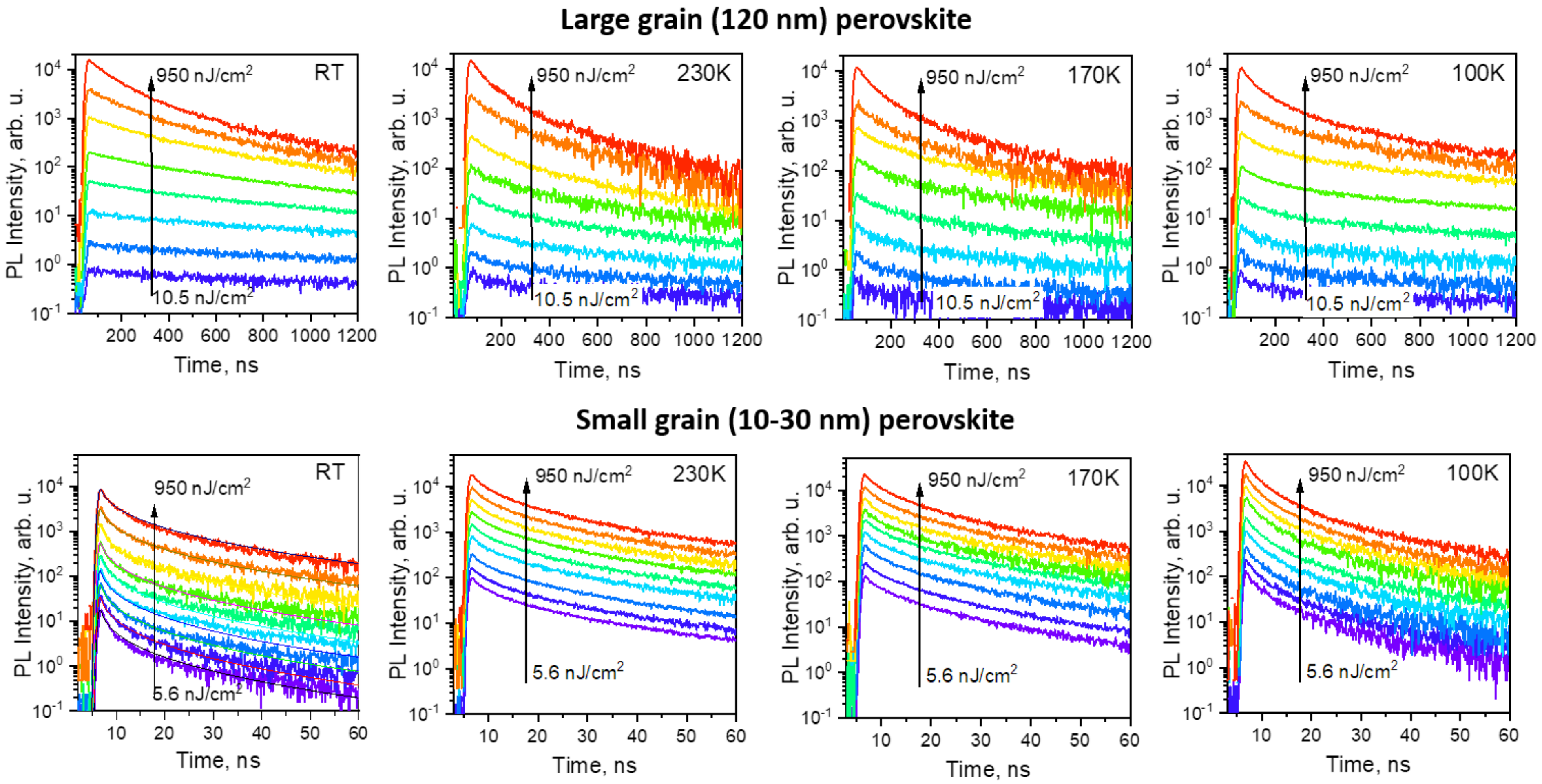

Figure S6. PL decay kinetics at different excitation intensities and different temperatures obtained for the large-grain and small-grain films.

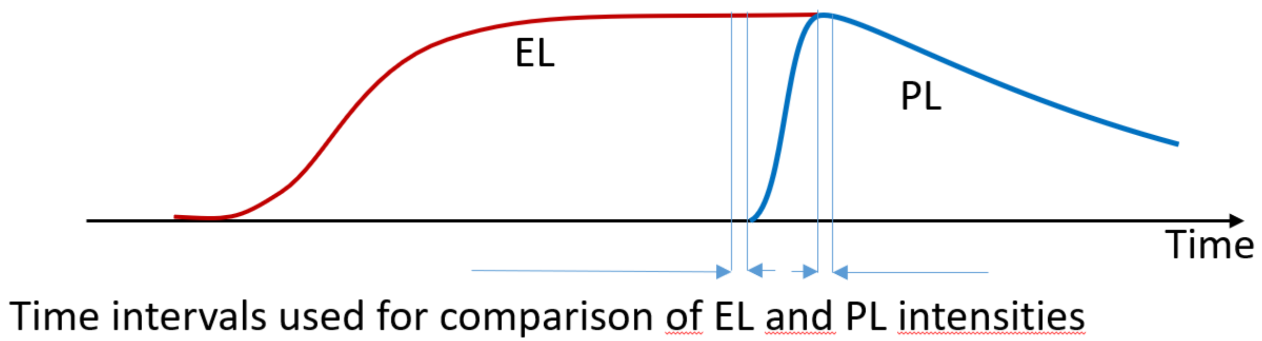

Figure S7. Time diagram of the comparison of PL and EL intensities and carrier densities.

Modeling of the PL kinetics by partly confined carrier (PPC) model.

We assume that perovskite grains form a lattice, and denote $n_e^{(i)}$ as the number of electrons at the i-th site and $n_h^{(i)}$ as the number of holes at the i-th site. We are interested in the photoluminescence signal, which is proportional to the mean number of electron and hole pairs at a single site, $PL \sim \langle n_e n_h \rangle$. Thus, we will be modeling the latter quantity.

The number or electrons at each site changes due equilibration (hopping between the sites), trapping (hopping to traps) and recombination. We assume that the process of recombination is much slower than the other two processes, thus it will be ignored for simplicity. Therefore, we can write the following equation for the number of electrons at i-th site:

$$\frac{\mathrm{d}}{\mathrm{d}t} n_e^i = -k_{\mathrm{eq}}(t) \cdot \left(n_e^i - \langle n_e \rangle\right) - k_{\mathrm{trap}}(t) \cdot n_e^i$$

Here $k_{\mathrm{eq}}(t)$ is the equilibration rate, $k_{\mathrm{trap}}(t)$ is the trapping rate and $\langle n_e \rangle$ is the mean number of electrons at one site. Equation for $n_h^{(i)}$ looks analogously. We assume time-dependent rates for equilibration and trapping,

$$k_{\mathrm{eq}}(t) = \begin{cases} k_0^{\mathrm{eq}}, & t < t_0; \\ k_0^{\mathrm{eq}} \left(\frac{t}{t_0}\right)^{-\alpha}, & t \geq t_0; \end{cases}$$

and

$$k_{\mathrm{trap}}(t) = \begin{cases} k_0^{\mathrm{trap}}, & t < t_0; \\ k_0^{\mathrm{trap}} \left(\frac{t}{t_0}\right)^{-\gamma}, & t \geq t_0. \end{cases}$$

Parameter t_0 is introduced to avoid divergence at $t = 0$. We set t_0 equal to 1 ps in all our calculations. Parameters α and γ are assumed to be less than 1.

The mean number of electrons at one site evolves via equation

$$\frac{\mathrm{d}}{\mathrm{d}t} \langle n_e \rangle = -k_{\mathrm{trap}}(t) \cdot \langle n_e \rangle,$$

which has a solution

$$\langle n_e \rangle = \begin{cases} \langle n_0 \rangle \exp(-k_0^{\text{trap}}), & t < t_0; \\ \langle n_0 \rangle \exp\left[-k_0^{\text{trap}}\left(t_0 + \frac{1}{1-\gamma}\left(\left(\frac{t}{t_0}\right)^{-\gamma} t - t_0\right)\right)\right], & t \geq t_0. \end{cases}$$

Here $\langle n_0 \rangle$ is the initial mean number of electrons at a single site, which is equal to the mean number of initial excitations, as each excitation is comprised by an electron and hole pair.

Meanwhile, the mean number of electron and hole pairs at a single site evolves via equation

$$\frac{\mathrm{d}}{\mathrm{d}t}\langle n_e h_h \rangle = -2k_{\text{eq}}(t) \cdot (\langle n_e n_h \rangle - \langle n_e \rangle \langle n_h \rangle) - 2k_{\text{trap}}(t) \cdot \langle n_e n_h \rangle.$$

This equation can be solved numerically. The initial condition is that at time zero $\langle n_e h_h \rangle = \langle n_0 \rangle + \langle n_0 \rangle^2$. Additional simplification results since $\langle n_e \rangle$ and $\langle n_h \rangle$ satisfy an identical equation with an identical initial condition, thus we can set $\langle n_e \rangle = \langle n_h \rangle$ for all times.

Assuming $\langle n_0 \rangle$ is known for each case (we used estimations described in the main text), the PL kinetics at all excitation intensities can be described by only four parameters: k_0^{eq}, k_0^{trap} , α and γ. Since our initial estimations for $\langle n_0 \rangle$ are not exact due to the assumed simplification, we introduce another parameter β which describes the ratio between $\langle n_0 \rangle_{\text{fitted}} = \beta \langle n_0 \rangle_{\text{estimated}}$ When calculating the PL kinetics, the obtained time dependence $\langle n_e h_h \rangle(t)$ was convolved with an instrument response function approximated by a Gaussian with FWHM $= 750$ ps. The calculated signal for the 950 nJ/cm^2 curve was normalized to the experimental PL kinetics, and other calculated curves were normalized using the same normalization factor. We have adjusted the parameter values by hand to match the overall behavior of the PL decays, considering that some of the amplitudes could be slightly off due to, *e. g.*, fluctuations in laser power. The obtained values for parameters were: $k_0^{\text{eq}} = (0.0345 \text{ ns})^{-1}$, $k_0^{\text{trap}} = (0.0882 \text{ ns})^{-1}$, $\alpha = 0.865$, $\gamma = 0.605$ and $\beta = 0.453$. All the PL curves were calculated using these parameter values.

Using our parameter values we can make some estimations of the carrier mobility and diffusion coefficient. The diffusion coefficient can be approximated by $D = L^2 \cdot k_{\text{eq}}$. Assuming that the distance between the grains is $L = 20$ nm, we obtain the value of $D = 0.12 \text{ cm}^2/\text{s}$ at times less than t_0. As we assume that k_{eq} is time-dependent, so is the diffusion coefficient, which has an identical form of time dependence. Evaluating the diffusion coefficient at $t = 1$ ns , we obtain $D = 3 \cdot 10^{-4} \text{ cm}^2/\text{s}$. We can evaluate the mobility using the Einstein relation, $\mu = qD/(k_B T)$. We obtain values $\mu = 4.6 \cdot \text{cm}^2/(\text{V} \cdot \text{s})$ for times less than t_0 and $\mu = 0.01 \cdot \text{cm}^2/(\text{V} \cdot \text{s})$ for $t = 1$ ns.

It is important to note that the obtained values for the carrier mobility and diffusion coefficient are rather rough estimations. The key point is that the parameter k_{eq} used in our model does not strictly coincide with the hopping rate between neighboring sites, which should be used in the estimation of D. Our parameter k_{eq} takes into account both forward and backward hopping and the number of neighboring sites. Nonetheless, the hopping rate should be of the same order as k_{eq}, thus our estimations should provide reasonable values.
